# 1-calcium phosphate-uracil, a synthesized pyrimidine derivative agent, has anti-proliferative, pro-apoptotic and anti-invasion effects on multiple tumor cell lines

**DOI:** 10.3892/mmr.2014.2489

**Published:** 2014-08-14

**Authors:** JING PENG, XINLIAN CHEN, QIAN HU, MEI YANG, HONGQIAN LIU, WEI WEI, SHANLING LIU, HE WANG

**Affiliations:** 1 Laboratory of Genetics, West China Institute of Women and Children’s Health, West China Second University Hospital, Sichuan University, Chengdu, Sichuan 610041, P.R. China; 2 Laboratory of Cell and Gene Therapy, West China Institute of Women and Children’s Health, West China Second University Hospital, Sichuan University, Chengdu, Sichuan 610041, P.R. China; 3 Department of Obstetrics and Gynecology, West China Second University Hospital, Sichuan University, Chengdu, Sichuan 610041, P.R. China; 4 Key Laboratory of Obstetrics, Gynecology, Pediatric Diseases and Birth Defects of the Ministry of Education, Sichuan University, Chengdu, Sichuan 610041, P.R. China

**Keywords:** 1-calcium phosphate-uracil, pyrimidine derivatives, apoptosis, invasion

## Abstract

1-calcium phosphate-uracil (1-CP-U), a synthetic pyrimidine derivative, has been documented to demonstrate a variety of different biological activities. However, the potency and mechanisms of this agent’s anti-cancer activity have not been elucidated to date. In the present study, the anti-cancer effects of 1-CP-U were examined in a range of *in vitro* assays. Different cell lines were treated with 1-CP-U at varied concentrations (0.7, 1.0, 1.4 μmol/l) for indicated durations. The cell proliferation was then examined by MTT assay. The cellular apoptotic effects were detected by Hoechst 33342 and Annexin V/propidium iodide staining, while the capacity of 1-CP-U on invasion and migration were examined by cell invasion and wound healing assays. The expression of matrix metalloproteinase proteins, as well as pro- and antiapoptotic proteins was detected by western blotting analysis. The results identified that 1-CP-U was able to inhibit the viability of SKOV3, HeLa, SMMC-7721 and A549 cell lines in a dose- and time-dependent manner, while it exerted only marginal toxic effects on non-cancerous cells. The IC_50_ concentration of 1-CP-U for tumor cell lines was ~1.0 μmol/l. The growth inhibition induced by 1-CP-U was accompanied by a broad spectrum of pro-apoptotic activities, in which different cell lines varied in their sensitivity to 1-CP-U. Meanwhile, the increased expression of the pro-apoptotic protein B-cell lymphoma-2 (Bcl-2)-associated X and a marked reduction of Bcl-2 levels were associated with increased 1-CP-U concentrations. Additionally, anti-migration and anti-invasion effects of 1-CP-U were evidently associated with the downregulation of matrix metalloproteinase proteins. Of note, it was observed that 1-CP-U significantly inhibited both the migration and invasion at a lower concentration, as compared with the dose required to achieve significant inhibition of apoptosis. These results indicated that 1-CP-U appeared to be a more effective inhibitor of cell migration and invasion, rather than of apoptosis. In conclusion, the present study was the first, to the best of our knowledge, to demonstrate the function of 1-CP-U in tumor proliferation, apoptosis and invasion with specific effects against cancer cells *in vitro*, suggesting 1-CP-U as a potential novel anticancer agent.

## Introduction

The increasing number and prevalence of neoplastic diseases, as well as their associated high mortality rates, have stimulated an unprecedented level of studies directed towards the development of novel antitumor drugs (1). Due to their compromised immunity, cancer patients during chemotherapy are highly susceptible to microbial infections. Co-administration of multiple drugs in the treatment of patients with neoplasms accompanied with low immunity may induce additional health problems, particularly in those whose kidney function is impaired. Furthermore, numerous chemotherapeutic agents have a number of unpleasant side effects, which may severely worsen the quality of life for the patient (2). Therefore, treatment with a single drug with low cytotoxicity as a monotherapy may be beneficial both therapeutically and economically.

Pyrimidine-containing compounds have been recently reported to possess a variety of anticancer effects (3,4). 1-calcium phosphate-uracil (1-CP-U) is a synthetic uracil derivative, which is a pyrimidine-containing compound and has been suggested to demonstrate a wide range of highly selective functions (5). It was hypothesized in the current study that 1-CP-U may enhance human immunity, regulate renal function and possess several pharmacological properties, including analgesic and antipyretic activities. In light of the above hypothesis, it was considered important to elucidate the anticancer effects of 1-CP-U, which, to the best of our knowledge, has not been studied previously, with the aim of identifying a more active and selective anticancer agent for the treatment of malignant neoplasms.

Apoptosis, or programmed cell death, is attenuated in numerous types of tumors that succeed in progressing to states of high-grade malignancy and resistance to therapy (6). A number of preclinical and clinical studies have demonstrated that the magnitude of induction of apoptosis is associated with tumor response to therapy (7) and that the disruption of the apotosis program may decrease treatment sensitivity (8). Loss of pro-apoptotic protein B-cell lymphoma (Bcl-2)-associated X protein (Bax) or overexpression of the anti-apoptotic protein Bcl-2 is frequently observed in tumor cells resistant to cancer therapy (9). Metastasis is a prominent limitation in the treatment of cancer, as the majority of the associated mortality is correlated with the disseminated disease rather than the primary tumor (10). During the process of metastasis formation, the degradation of the extracellular matrix (ECM) by proteases, including matrix metalloproteinases (MMPs), has an important role. The expression and activity of MMPs are enhanced in almost all types of human cancer, and this correlates with advanced tumor stage and shortened survival (11).

The present study has revealed for the first time, to the best of our knowledge, a broad spectrum of anti-cancer activities of 1-CP-U against a number of different human tumor cell lines. The obtained data indicate that 1-CP-U possesses a variety of effects on cancer cell proliferation, apoptosis, migration and invasion *in vitro*.

## Materials and methods

### Cell lines and culture

Human cervical cancer cell lines HeLa and CaSki, human ovarian cancer cell line SKOV3, human hepatocellular carcinoma cell line SMMC-7721, human lung adenocarcinoma cell line A549, human colorectal carcinoma cell line LS174T, normal lung fibroblasts MRC-5 and human embryonic kidney (HEK-293) cells were obtained from the American Type Culture Collection (Manassas, VA, USA). SKOV3, Hela, SMMC-7721, A549, LS174T and HEK-293 cells were maintained in Dulbecco’s modified Eagle’s medium (DMEM; Gibco-BRL, Carlsbad, CA, USA) supplemented with 10% heat-inactivated fetal bovine serum (FBS; HyClone, Logan, UT, USA) and 100 U/ml penicillin and streptomycin (Wuhan Boster Biological Technology., Ltd., Wuhan, China). CaSki cells were cultured in RPMI-1640 medium (HyClone) supplemented with 10% FBS, and 100 U/ml penicillin and streptomycin. The MRC-5 cell line was maintained in DMEM supplemented with 15% FBS and 100 U/ml penicillin and streptomycin. All of the cells were cultured at 37°C in a 5% CO_2_ incubator.

### Chemotherapeutic drug and antibodies

**Figure 1:**
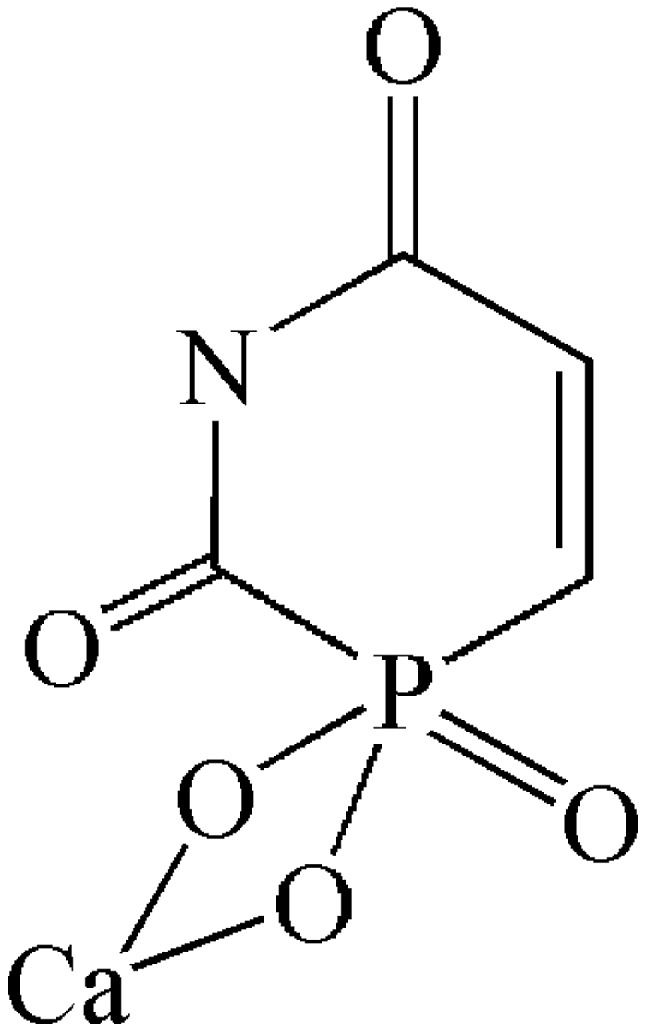
Chemical structure of 1-calcium phosphate-uracil with a molecular weight of 216.1276 g/mol.

1-CP-U, synthesized according to the reported procedure (5), was generously provided by Ms. Ning Qizhi, who originally synthesized the agent. The chemical structure of 1-CP-U is demonstrated in Fig. 1. A total of 1.0 g of 1-CP-U crystal was weighed, totally dissolved it in ultrapure water facilitated by a 0.25 mol/l hydrochloric acid solution, and then the pH was adjusted to 4.0 by adding 0.25 mol/l sodium hydroxide solution. Calculating the concentration of the stock solution as 63.39 mM, it was diluted to the required concentrations in conditional medium and then stored at 4°C. The antibodies were as follows: Polyclonal rabbit anti-human Bax, MMP-2 and MMP-9, monoclonal mouse anti-human Bcl-2, (Wuhan Boster Biological Technology., Ltd.; A0315-2, BA0569, BA0573 and BM0200, respectively), and β-actin was purchased from Zhongshan Golden Bridge Biotechnology Co., Ltd., (Beijing, China; TA-09). The goat anti-mouse or anti-rabbit secondary antibodies were from Zhongshan Golden Bridge Biotechnology (ZB-2305 and ZB-2301).

### Cell proliferation assay

The effects of 1-CP-U on the different cell lines were determined by the MTT assay as described previously (12). Briefly, 5,000 cells were incubated in triplicate on a 96-well plate under normal culture conditions overnight. MRC-5, HEK-293, LS174T and CaSki cell lines were then treated with 1.0 μmol/l of 1-CP-U while SKOV3, HeLa, SMMC-7721 and A549 were treated with different concentrations of 1-CP-U (0.7, 1.0 and 1.4 μmol/l). The control group was incubated with an identical volume of drug-free dilute solution. Following this, 5 mg/ml MTT solution (Beyotime Institute of Biotechnology, Haimen, China) was added every 24 h to examine the cell viability on each day. Following 4 h incubation with MTT at 37°C, 150 μl dimethyl sulfoxide (DMSO) was added into each well to dissolve the formed crystals. Using the DMSO without tumor cells as a blank control, the optical density (OD) at 595 nm was measured by a 96-well microplate reader (Model-680; Bio-Rad, Hercules, CA, USA). The cell viability was expressed as a percentage according to the following equation: OD of the experiment samples / OD of the control × 100%. From these results, a dose- and time-dependent curve of the 1-CP-U-treated tumor cell lines was generated. The cytotoxic effects of 1-CP-U were expressed as the 50% inhibitory concentration (IC_50_) calculated by SPSS 13.0 (SPSS, Inc., Chicago, IL, USA).

### Apoptosis analysis

The rate of apoptosis was analyzed by two different methods. Firstly, chromatin staining with Hoechst 33342 was performed to detect nuclear condensation. Briefly, tumor cell cultures were seeded in six-well plates and treated with different concentrations of 1-CP-U for 48 h. The apoptotic morphology of the cells was evaluated by Hoechst 33342 (C1025; Beyotime Institute of Biotechnology) following the manufacturer’s instructions. Secondly, the Annexin V-fluorescein isothiocyanate (FITC) assay was employed to measure the percentage of the apoptotic cells under 1-CP-U treatment. Following treatment with 1-CP-U, the cells were trypsinized, collected and washed with phosphate-buffered saline (PBS). After using the Annexin V-FITC Apoptosis Detection kit (KGA107; KeyGen Biotech Co., Ltd., Nanjing, China) which contained propidium iodide (PI), the stained cells were analyzed using a flow cytometer (BD FACS AriaII; BD Biosciences; Franklin Lakes, NJ, USA).

### Wound healing migration assay

The cell motility was measured by a wound healing assay as described previously (10). Initially, equal numbers of tumor cells were allowed to grow in six-well plates overnight. The next day, the cells were scraped with pipette tips and washed with PBS. The cells were then treated with or without the 1-CP-U under starvation to inactivate cell proliferation. The cells migrated into the wound surface and the average distance of migrating cells was determined under an Eclipse TS100 inverted microscope (Nikon Corporation, Tokyo, Japan) at the designated time-points.

### Cell invasion assay

The invasion assay was performed using Boyden chambers consisting of a 24-well Millicell inserts and a membrane filter (8 μm pore size) (Merck Millipore, Chengdu, China) as described previously (13). Briefly, the upper chamber of the Boyden chamber was coated with 40 μl Matrigel (1 mg/ml), and left to solidify at 37°C for 30 min. The bottom chambers were filled with 600 μl DMEM containing 20% FBS. The top chambers were seeded with 2×10^5^ cells in the absence or presence of 0.7 μmol/l 1-CP-U. Following 24 h of incubation, the cells on the top surface of the filter were scraped, and those spread on the bottom sides (invasive cells) were fixed with cold 4% paraformaldehyde and stained with eosin (AR1180-2; Wuhan Boster Biological Technology., Ltd.) alone. Images of the cells were captured with a the inverted microscope and then quantified.

### Immunoblot analyses

The HeLa cells were treated with different concentrations of 1-CP-U for 48 h. Whole cellular protein was extracted with radioimmunoprecipitation assay buffer (P0013B; Beyotime) containing 1 mM phenylmethanesulfonylfluoride (ST506; Beyotime). The mix was centrifuged at 12,000 × g for 5 min at 4°C. Following this, the protein concentration was determined by the bicinchoninic acid method (P0012S; Beyotime) according to the manufacturer’s instructions using a Bio-Rad assay. After heating to 95°C for 5 min, a total of 25 μg protein was separated by 8% Tris-HCl SDS-PAGE, and then transferred to an polyvinylidene fluoride membrane (Bio-Rad; 162-0177) using wet transfer apparatus (Bio-Rad; MiniProtean Tetra). The membrane was incubated in blocking solution [5% non-fat milk in Tris-buffered saline containing 0.1% Tween-20 (TBS/T)] for 1 h with gentle rocking at room temperature. The primary antibody was diluted and the membrane was incubated at 4°C overnight with the following antibodies: Bax (1:600), Bcl-2 (1:600), MMP-2 (1:400) and MMP-9 (1:400). Following washing for 15 min in TBS/T three times, the membranes were incubated with the secondary antibodies (1:5,000) for 1 h. After washing, visual detection was performed using the enhanced chemiluminescence method (WBKLS0100; Merck Millipore).

### Statistical analysis

The results are expressed as the mean ± standard deviation and statistically compared by one-way analysis of variance test or unpaired Student’s t-test in different experiments. P<0.05 was considered to indicate a statistically significant difference.

## Results

### 1-CP-U treatment inhibits the growth of human tumor cell lines

**Figure 2:**
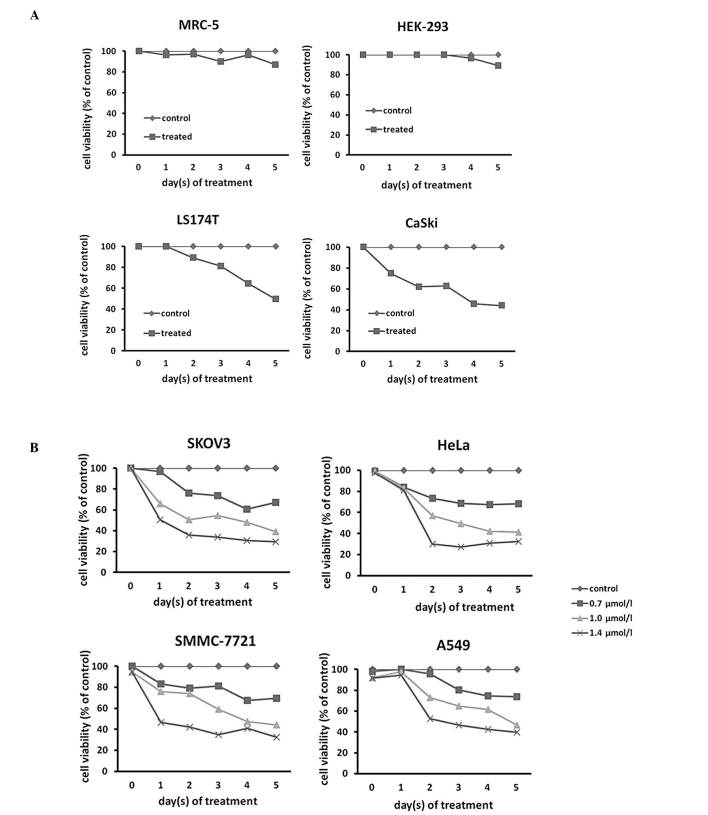
(A and B) 1-CP-U-induced cytotoxicity. A total of 5,000 cells were seeded in triplicate in 96-well plates and then incubated with 1-CP-U for five days. The LS174T, CaSki, MRC-5 and HEK-293 cells were subject to treatment with 1-CP-U (1.0 μmol/l). The SKOV3, HeLa, SMMC-7721 and A549 cells were treated with increasing concentrations of 1-CP-U (0.7, 1.0 and 1.4 μmol/l). The cell viability was then analyzed by MTT assay. Data are presented as the mean ± standard deviation of three separate experiments. 1-CP-U, 1-calcium phosphate-uracil.

To determine whether 1-CP-U was able to exert cytotoxic effects on human tumor cells, a panel of human tumor cell lines, including LS174T, CaSki, SKOV3, HeLa, SMMC-7721 and A549, were treated with 1-CP-U. The results demonstrated that treatment with 1.0 μmol/l of 1-CP-U resulted in >50% cell death following five-day treatment in the LS174T and CaSki populations (Fig. 2A). Next, the impact of 1-CP-U treatment (0.7, 1.0 and 1.4 μmol/l for five days) on SKOV3, HeLa, SMMC-7721 and A549 cell lines was investigated and it was identified that 1-CP-U concentration-dependently suppressed proliferation in the four examined tumor cell lines (Fig. 2B). The IC_50_ of 1-CP-U for the SKOV3, HeLa, SMMC-7721 and A549 cell lines was 0.909, 0.941, 1.068 and 0.971 μmol/l following five days of exposure, respectively. There was a significant difference between the viability of the treated cell lines and that of the control population following an incubation period of at least 24 h (P<0.05). To determine the selectivity of 1-CP-U to tumor cells, the normal MRC-5 and HEK-293 cells were treated with 1.0 μmol/l 1-CP-U, which resulted in only a marginal toxic effect (Fig. 2A), indicating that this effect was likely specific to cancer cells.

### 1-CP-U induces apoptosis in tumor cells

**Figure 3:**
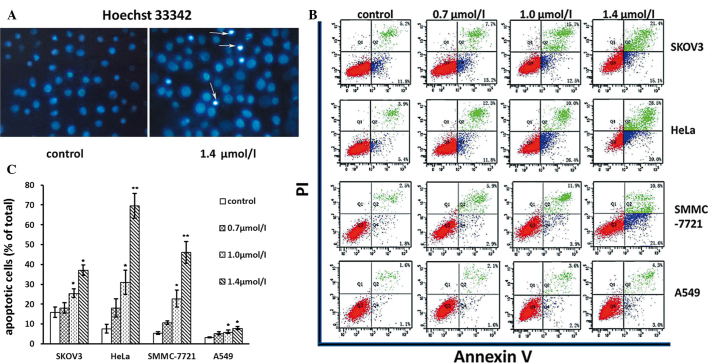
Effects of 1-CP-U on apoptosis. (A) Tumor cells were treated with various concentrations of 1-CP-U (0.7, 1.0 and 1.4 μmol/l) for 48 h, and then stained with Hoechst 33342 dye. The strong blue signals indicate apoptotic cells of SMMC-7721 with nuclear chromatin condensation and the formation of nuclear fragments and apoptotic bodies (arrows; magnification, ×40). (B) Tumor cells were stained with Annexin V/PI dye at the indicated time. Analyses were conducted on 10,000 cells in each trail. The cells in the early stages of apoptosis were only Annexin V-FITC positive. The cells in the late stage of apoptosis and those moving toward secondary necrosis stained positive with both Annexin V-FITC and PI (Q3, live cells; Q4, early stages of apoptosis; Q2, late stage of apoptosis and secondary necrosis); (C) The percentage of Annexin V positive cells representing apoptosis in (B) was quantified and statistically analyzed as the mean ± standard deviation. ^*^P<0.05; ^**^P<0.001 vs. controls. 1-CP-U, 1-calcium phosphate-uracil; PI, propidium iodide; FITC, fluorescein isothiocyanate.

Following observing the decline in cell viability caused by 1-CP-U, particularly at higher concentrations, the induction of apoptosis by 1-CP-U was assessed. Firstly, tumor cells were cultured for 48 h with or without 1-CP-U, and apoptosis-specific morphology was visualized by Hoechst 33342 staining. Condensed chromatin and apoptotic bodies were identified in the 1-CP-U-treated cells, as determiend by fluorescence microscopy (Fig. 3A). Secondly, flow cytometric analysis was performed following staining of the cells with Annexin-V-FITC plus PI (Fig. 3B). The apoptotic rates in the 1.0 μmol/l 1-CP-U group were 25.50±4.33, 31.00±12.04, 22.87±8.57 and 6.03±1.76 for SKOV3, HeLa, SMMC-7721 and A549 cell lines, respectively. The apoptotic rates in the 1.4 μmol/l 1-CP-U groups were 37.17±5.13, 69.57±12.63, 46.07±11.01 and 7.97±1.61%, respectively. The apoptotic rates in the two treated groups were significantly higher than those in the control group (15.93±5.18, 7.60±4.31, 5.43±1.40% and 3.23±0.47%, respectively; P<0.05), enhanced with increasing doses of 1-CP-U (Fig. 3C). Among the four cell lines exposed to 1-CP-U, the number of apoptotic cells in the SKOV3, HeLa and SMMC-7721 populations increased markedly, particularly in the HeLa cells (9.15-fold at 1.4 μmol/l 1-CP-U treatment as compared with the corresponding control group), while a comparably modest increase was detected in the A549 cells (2.47-fold at 1.4 μmol/l 1-CP-U treatment as compared with the corresponding control group), suggesting that different cell lines varied in their sensitivity to 1-CP-U.

### 1-CP-U inhibits tumor cell migration and invasion in vitro

**Figure 4:**
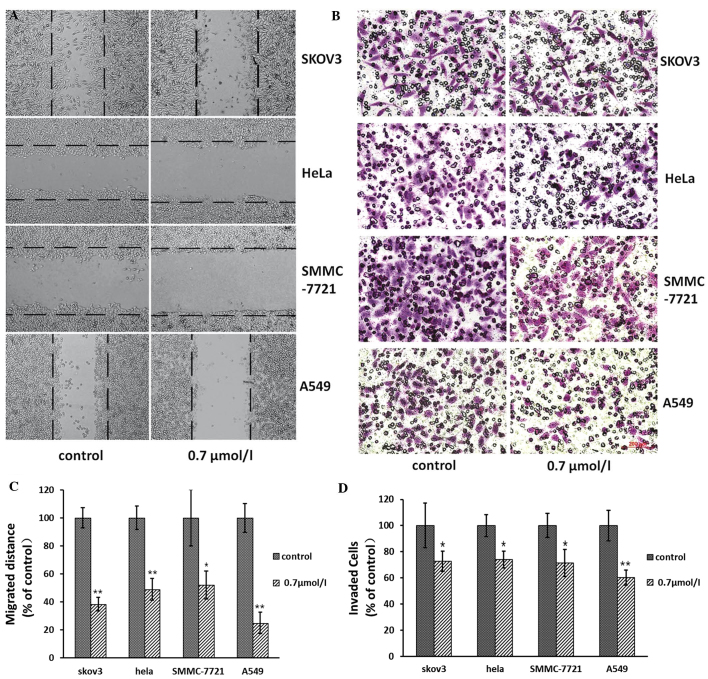
1-CP-U inhibits migration and invasion of tumor cells. (A) Wound healing assay. Tumor cells were scratched with a pipette tip and then treated with 0.7 μmol/l 1-CP-U. The cells migrated into wound surface and the average distance of migrating cells was determined by phase-contrast microscopy (magnification, ×20); (B) Cell invasion assay. The cells in the lower surface of the Borden chamber were stained with eosin and images were captured under the microscope (magnification, ×40); (C) migratory distance in (A) was quantified by manual measuring. The ratio of the migration distance in the treatment group and that in the corresponding control group was calculated. (D) invasive tumor cells in (B) were quantified by manual counting. The ratio of the number of invaded tumor cells in the treatment group to that in the corresponding control group was quantified and presented in the bar chart. Data are presented as the mean ± standard deviation of three separate experiments. ^*^P<0.05; ^**^P<0.001 vs. controls. 1-CP-U, 1-calcium phosphate-uracil.

The wound healing assay was performed in the presence or absence of 1-CP-U in the SKOV3, HeLa, SMMC-7721 and A549 cells. The tumor cells were imaged following treatment for 0 and 24 h at the same marked site. The migration distance, which was the difference in wound width at both time-points (0 and 24 h following treatment), was measured (Fig. 4A). The effect of 1-CP-U on the invasion of tumor cells was measured by Boyden chamber invasion assay (Fig. 4B). The ratio of the migration distance in the treatment group to that in the corresponding control group was 38.26, 48.95, 52.08 and 24.84% for SKOV3, HeLa, SMMC-7721 and A549 cells, respectively (Fig. 4C). Of note, 1-CP-U markedly inhibited tumor cell migration. The number of SKOV3, HeLa, SMMC-7721 and A549 cells passing through the Matrigel in the negative control group (35±12, 30±5, 32±6 and 39±9, respectively) was significantly higher than that in the 0.7 μmol/l 1-CP-U group (13±4, 10±2, 14±3 and 17±5, respectively). The ratio of the number of invaded tumor cells in the treatment group to that in the corresponding control group was calculated and presented in a bar chart (Fig. 4D). The results indicated that 1-CP-U markedly suppressed the cell invasion through the Matrigel.

### 1-CP-U affected expression of apoptotic and MMP proteins

**Figure 5:**
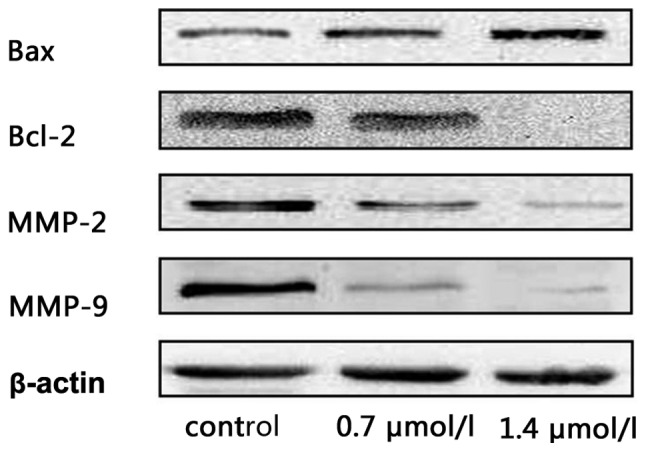
1-CP-U downregulates MMP-2 expression in tumor cells. HeLa cells were treated with 1-CP-U for 48 h at different concentrations (control, 0.7 and 1.0 μmol/l). The expression of Bax, Bcl-2 and MMP proteins were detected by western blotting analysis. 1-CP-U, 1-calcium phosphate-uracil; MMP, matrix metalloproteinase; Bcl-2, B-cell lymphoma 2; Bax, Bcl-2-associated X.

The finding that 1-CP-U induced apoptosis and inhibited the invasion of tumor cells prompted us to examine its effect on apoptotic and MMP proteins using western blotting analysis. HeLa cells were treated with 1-CP-U for 48 h at different concentrations (0, 0.7 and 1.0 μmol/l). Increased expression levels of the pro-apoptotic protein Bax and a marked reduction of Bcl-2 levels were also associated with increased 1-CP-U concentrations. Meanwhile, 1-CP-U suppressed the secretion of MMP-2 and MMP-9 in a dose-dependent manner (Fig. 5).

## Discussion

A number of recent studies have revealed that pyrimidine derivatives demonstrated efficient anticancer activities (14). For example, pyrrole and pyrrolo[2,3-d]pyrimidine derivatives have aroused notable attention as potent anticancer agents (15). Pyrimidine-2,4,6-triones have been identified as a new effective and selective class of MMP inhibitors (16). 1-CP-U is a synthetic pyrimidine derivative (5). Compared with polysubstituted pyrimidines mentioned previously, 1-CP-U has a simpler chemical structure, which increases its cost-effectiveness as well as its ease of production and administration. However, the structure of 1-CP-U remains to be confirmed by electrospray ionization-mass spectrometry, and in addition, ^1^H and ^13^C nuclear magnetic resonance spectral analysis and the purity determination should be investigated in future studies. Thus far, few of its bioactivities against human tumor types have been reported.

In the present study, a variety of biological responses initiated by 1-CP-U *in vitro* were investigated for the first time to the best of our knowledge. Initially, the effects of 1-CP-U on tumor cell proliferation were investigated. 1-CP-U effectively induced growth inhibition in cultured SKOV3, HeLa, SMMC-7721 and A549 cells, with IC_50_ values of ~1.0 μmol/l (Fig. 2B). Additionally, whether 1-CP-U may affect the viability of non-cancerous cells was examined. The data obtained demonstrated that 1-CP-U exhibited low cytotoxicity on the healthy MRC-5 and HEK-293 cell lines at the concentration of 1.0 μmol/l (Fig. 2A), suggesting that cell proliferation inhibition caused by 1-CP-U is an effect specific to cancer cells.

It is well established that the majority of anticancer agents induce apoptosis (7). Therefore, following detecting a decline in cell viability caused by 1-CP-U, the apoptosis induced by 1-CP-U was assessed using Hoechst 33342 staining and flow cytometric analysis (Fig. 3A and B). It was noted that 1-CP-U at 1.0 and 1.4 μmol/l induced significant levels of apoptosis in SKOV3, HeLa, SMMC-7721 and A549 cell lines (Fig. 3C). Additionally, 1-CP-U initiated only a modest increase in the apoptotic rate in A549 cells compared with that in the SKOV3, HeLa and SMMC-7721 cell lines. Possibly heterogeneous tumor cell populations exhibit different drug sensitivities and are also susceptible to more than one type of cell death (8). The activation of the pro-apoptotic proteins Bax and Bcl-2 homologous antagonist killer (Bak) results in the translocation of Bax/Bak from the mitochondria to the cytoplasm, thereby promoting Bax/Bak oligomerization, which leads to the release of a number of small molecules (17). This is inhibited by the anti-apoptotic proteins Bcl-2 and Bcl-2 extra large protein (Bcl-xL), which are major inhibitors of apoptotic cell death (18). In the present study, 1-CP-U increased the expression levels of Bax while suppressing the levels of Bcl-2 in a dose-dependent manner (Fig. 5).

Migration and invasion of cancer cells are key steps in tumor metastasis (19). The results revealed that 0.7 μmol/l 1-CP-U significantly inhibited both the migration and invasion of the SKOV3, HeLa, SMMC-7721 and A549 cell lines (Fig. 4). MMPs are a family of zinc-dependent endopeptidases first described almost half a century ago (20). They have a crucial role in ECM degradation, associated with tissue repair, cancer cell invasion, metastasis and angiogenesis (21,22). Among several MMPs, MMP-2 and -9 have been demonstrated to be critical factors in tumor invasion (23), which is secreted by tumor cells as a pro-enzyme (pro-MMP-2) and activated in the extracellular milieu to execute their proteolytic activity, then accordingly enables cells to invade into the target organ and develop tumor metastasis (24,25). A previous study demonstrated that increased expression of MMPs (26) is linked with lymphatic invasion and lymph node metastases. Inhibition of MMPs attenuated angiogenesis and lymphangiogenesis, and reduced lymph node metastasis (27). In the present study, western blot analysis identified that treatment with 1-CP-U inhibited the expression of MMP proteins in a dose-dependent manner in the HeLa cells (Fig. 5). The results indicated that MMP-2 may be a downstream target of 1-CP-U.

Of note, it was observed that 1-CP-U significantly inhibited the migration and invasion at a lower concentration (0.7 μmol/l) compared with the dosage of 1-CP-U required to achieve significant inhibition of apoptosis (1.0 and 1.4 μmol/l). These results revealed that 1-CP-U appeared to be more effective at inhibiting cell migration and invasion than inducing apoptosis, suggesting that the anti-migration and anti-invasion functions of 1-CP-U may have more clinical potential over its pro-apoptotic activity.

In conclusion, the present study demonstrated that 1-CP-U exhibited anti-cancer activity on a panel of SKOV3, HeLa, SMMC-7721 and A549 cell lines, comprising cell proliferation, apoptosis, migration and invasion. The antitumor effects suggested that 1-CP-U may be considered as a candidate anticancer agent against a broad spectrum of tumor types, which deserves further investigation in order to examine its biological activities and molecular mechanisms in a clinical setting.
